# Real-world treatment and fracture incidence in postmenopausal women with severe osteoporosis at high risk of fracture: a retrospective claims data analysis

**DOI:** 10.3205/000302

**Published:** 2021-12-23

**Authors:** Antje Mevius, Tanja Heidbrede, Patrick Gille, Hans Derk Pannen, Thomas Wilke

**Affiliations:** 1 Institute for Pharmacoeconomics and Medication Logistics (IPAM) e.V., University of Wismar, Germany; 2 UCB Pharma, Monheim, Germany

**Keywords:** postmenopausal osteoporosis, high risk of fractures, real-world treatment, guideline adherence

## Abstract

**Background:** Osteoporosis (OP) and its associated fractures have a significant impact on patients’ quality of life and are impacting their morbidity and mortality. For OP patients at high risk of fracture, guidelines recommend a pharmacological OP treatment. The aim of this study was to describe the real-world medication treatment of postmenopausal women with severe OP at high risk of fracture, their risk to experience a new fracture after having at least one previous fracture, and to assess the associated healthcare resource use (HCRU).

**Methods:** This retrospective cohort study was based on anonymized German claims data (AOK PLUS). All included OP patients were female, ≥55 years old, and had a vertebral and/or femoral fracture. We conducted a cross-sectional analysis in 2018 and a longitudinal analysis, starting with an incident vertebral/femoral fracture (after or simultaneously with the first observed OP diagnosis). In both analyses, patient characteristics, rate of new incident fractures, OP treatment patterns, and HCRU associated with the treatment of patients were investigated.

**Results:** In the cross-sectional setting, 12,180 patients with a mean age of 83.59 years were observed. Of these patients, 14.30% sustained at least one new incident fracture and 34.54% received a pharmaceutical OP treatment during 2018. In this year, 58.50% of the patients had at least one OP-related outpatient visit, and 26.35% had a fracture-related visit. In 160 patients (1.31%), at least one OP-related hospitalization was documented, and in 1,293 patients (10.62%) a fracture-related hospitalization in 2018.

In the longitudinal setting, 10,323 patients with a mean age of 83.22 years were included. Of these, 18.96% experienced at least one new incident fracture within the first 12 months after the index fracture, and in total 30.85% in the entire follow-up period (mean 2.03 years). During the 12-month baseline period, 22.12% of the patients received an OP treatment. Three months after the index fracture, the proportion of treated patients remained at 22.30%. During the total follow-up time, 35.54% were prescribed with an OP treatment.

**Conclusion:** We observed a considerable proportion of untreated patients and a high rate of subsequent fractures. The awareness for a proper risk assessment and the appropriate use of available treatments should be increased.

## Zusammenfassung

**Hintergrund:** Osteoporose (OP) und die damit verbundenen Frakturen haben einen signifikanten Einfluss auf die Lebensqualität von OP-Patienten und beeinflussen ebenso deren Morbidität und Mortalität. Leitlinien empfehlen für OP-Patienten mit hohem Frakturrisiko eine spezifische medikamentöse Therapie. Das Ziel dieser Studie war es, die Behandlungspraxis und das Risiko erneuter Frakturen von postmenopausalen Frauen mit manifester OP mit deutlich erhöhtem Frakturrisiko zu beschreiben sowie die damit verbundenen Ressourcenverbräuche zu ermitteln.

**Methoden:** Diese retrospektive Kohortenstudie basierte auf anonymen Krankenkassendaten (AOK PLUS). Sämtliche eingeschlossenen OP-Patienten waren weiblich, ≥55 Jahre alt und erlitten bereits eine vertebrale oder femorale Fraktur. In der Studie wurden zwei verschiedene Analysen durchgeführt: eine Querschnittsanalyse für das Jahr 2018 und eine Längsschnittanalyse beginnend mit der Indexfraktur (nach erster beobachteter OP-Diagnose in 2015–2017). In beiden Analysen wurden die Patientencharakteristika, die Rate neuer Frakturen, die medikamentöse OP-Behandlung sowie die assoziierten Ressourcenverbräuche untersucht.

**Ergebnisse:** In der Querschnittsanalyse wurden 12.180 Patientinnen mit einem durchschnittlichen Alter von 83,59 Jahren beobachtet. Von diesen Patientinnen erlitten in 2018 14,30% mindestens eine neue Fraktur und 34,54% erhielten eine medikamentöse OP-Behandlung. Im Beobachtungszeitraum besuchten 58,50% OP-bezogen einen ambulanten Arzt, 26,35% der Arztbesuche waren frakturbezogen. 160 (1,31%) bzw. 1.293 Patientinnen (10,62%) hatten 2018 einen stationären Aufenthalt mit OP- oder Fraktur-Bezug (primäre Diagnose).

In die Längsschnittanalyse wurden 10.323 Patientinnen mit einem Durchschnittsalter von 83,22 Jahren eingeschlossen. Innerhalb der ersten 12 Monate nach der Indexfraktur erlitten 18,96% mindestens eine weitere neue Fraktur, im gesamten Beobachtungszeitraum (Mittelwert 2,03 Jahre) 30,85%. In den 12 Monaten vor der Indexfraktur erhielten 22,12% der Patientinnen eine medikamentöse OP-Behandlung; 3 Monate nach dem Index lag der Anteil der Behandelten fast unverändert bei 22,30%. Während des gesamten Beobachtungszeitraums erhielten insgesamt 35,54% der Patientinnen OP-Medikamente.

**Schlussfolgerung:** In der Studie wurden ein beträchtlicher Anteil unbehandelter Patientinnen und eine hohe Rate an Folgefrakturen beobachtet. Das Bewusstsein für eine leitliniengerechte Risikobewertung und den zweckmäßigen Einsatz der verfügbaren Behandlungsoptionen sollte verbessert werden.

## Introduction

Osteoporosis (OP) is defined as a systemic skeletal disease characterized by low bone mass and microarchitectural deterioration of bone tissue, leading to an increase in bone fragility and susceptibility to fracture. Clinical manifestations of OP primarily include fractures of the hip, spine, distal forearm, and proximal humerus. OP and its associated fractures have a significant impact on patients’ quality of life and are impacting their morbidity and mortality markedly [1], [2], [3], [4], [5], [6], [7]. Especially vertebral and femoral fractures have shown to have a considerable impact on mortality and quality of life of affected patients [4], [8], [9], [10], [11]. The specific fracture risk of OP patients depends mainly on their age, previous fractures, their comorbidities as well as OP-related clinical characteristics. In addition, women are at particular risk of osteoporotic fractures, as accelerated bone loss occurs after menopause [12], [13].

Thresholds recommended for decision-making regarding initiation of a pharmacological OP treatment are based on probabilities for major osteoporotic and in particular hip and vertebral fractures [3], [14], [15]. Specifically patients with a prior proximal femoral or vertebral fracture can be considered as high-risk patients, so a respective treatment without the need for further assessment is recommended [3], [15]. For all postmenopausal women with OP at high risk of fracture, oral bisphosphonates are recommended as initial treatment according to the EFFORT/EULAR recommendations [14]. In women intolerant to oral bisphosphonates (or in those for whom these are contraindicated), intravenous bisphosphonates, denosumab, bazedoxifen, or raloxifene are appropriate alternatives. Teriparatide is only recommended for patients at very high risk of fracture, as stated in German and European guidelines and the summary of product characteristics [3], [14], [15], [16]. Additionally, the new agent romosozumab has been recently approved by EMA for the treatment of severe OP in postmenopausal women at increased risk of fracture [17]. Use of estrogens as OP prevention is recommended only in above women intolerant or contraindicated to any other therapy alternatives [3], [15].

So far, it is unknown to what extent the above guideline recommendations are implemented in clinical practice. Therefore, the aim of this study was to describe the German real-world treatment of postmenopausal women with severe OP at high risk of fracture, their risk to experience a new fracture after having at least one previous fracture, and to assess the associated healthcare resource use (HCRU).

## Methods

### Study design

This retrospective cohort study was based on anonymized claims data provided by AOK PLUS, a regional German statutory health insurance fund that represents, with 4.7% (3.4 M) of the statutorily insured German population, one of the biggest sickness funds in Germany. The dataset contained data on patients’ demographics (age, gender, level of care, receipt of a pension, date of death), outpatient treatment (visits to general practitioners (GPs) and specialists identified via physician code (“Arztgruppenschlüssel”, AGS code)) and related diagnosis codes, inpatient treatment (hospitalizations, diagnostic procedures, main diagnoses, length of stay), and outpatient medication prescriptions (anatomic therapeutic classification (ATC) codes, date of prescription, daily defined dose [DDD]).

### Study population

All included patients were female, were continuously insured by the sickness fund from 01/01/2014 until 31/12/2018 or death (whatever occurred first) and received at least one inpatient or one confirmed outpatient OP diagnosis (ICD-10 codes M80.-/M81.-) between 01/01/2015–31/12/2017.

The analysis was targeting postmenopausal women only. Since there is no specific code identifying the postmenopausal condition, an age proxy was used. Different studies published a mean/median age of natural menopause between 49 and 54 years, depending on the geographical region [18], [19], [20]. In a conservative consideration, the age threshold indicating postmenopausal cases was defined as age ≥55 years. Consequently, women aged below 55 years at the date of the first observed OP diagnosis were excluded from the analysis sample.

Furthermore, the study aimed to address female OP patients being at high risk of fracture only, which was assumed after the occurrence of a vertebral or femoral fracture during the 12-month pre-index period or at the index date, respectively (01/01/2018 for the cross-sectional analysis sample/date of incident fracture for the longitudinal analysis sample). Therefore, patients were included only if they received at least two outpatient or one outpatient plus one inpatient secondary diagnosis, or one inpatient main diagnosis of a vertebral and/or femoral fracture (ICD-10 codes: S12.-/S22.-/S32.-/S72.-/T08.-) that were documented after or at the date of the first OP diagnosis.

### Study outcomes and analyses

Identified patients were observed based on two different approaches. First, we conducted a cross-sectional analysis in 2018 to observe the treatment of all severe OP patients at high risk of fracture in a calendar year. Patients fulfilling above inclusion criteria who were still alive at 01/01/2018 were observed until 31/12/2018 or until death (whatever came first); note that patients who experienced already one or more additional fractures until 01/01/2018 after their incident fracture (between 01/01/2015–31/12/2017) were included in this sample. Second, a longitudinal analysis of target patients was performed to observe treatment patterns and potential treatment changes over time, starting with their incident vertebral/femoral fracture date (between 01/01/2015–31/12/2017) until 31/12/2018 or death (whatever came first). Patients were excluded from the longitudinal analysis if they had any fracture (ICD-10 codes: S12.-/S22.-/S32.-/S42.-/S52.-/S62.-/S72.-/S82.-/T08.-) in the observable period before the index event.

In both analyses, the following outcomes were investigated: patient characteristics, rate of new incident fractures after the index fracture, OP treatment patterns, and observed HCRU associated with the treatment of patients.

#### Patient characteristics

Patient characteristics were descriptively analyzed for both samples, based on the respective index date or a 12-month pre-index period. In addition to age, the proportion of patients with any level of care, the frequency of cardiovascular events, the most frequently observed comorbidities and any previous non-OP drug treatments were observed. For all categorical variables, the number and percentage of patients in each category were reported. Summary statistics, including mean and standard deviation (SD), were applied for all continuous variables.

#### New incident fractures after a first fracture

New incident fractures were defined as any fractures during the follow-up period as long as the same fracture (the same ICD-10 code) was not documented in the previous 12 months. Generally, the following events were considered for identification of new incident fractures:

- Vertebral/femoral fractures: Fractures of cervical vertebra and other parts of neck (ICD-10 S12.-), of rib(s), sternum and thoracic spine (ICD-10 S22.-), of lumbar spine and pelvis (ICD-10 S32.-), of femur (ICD-10 S72.-), of spine, level unspecified (ICD-10 T08.-).
- Other fractures: Fractures of shoulder and upper arm (ICD-10 S42.-), of forearm (ICD-10 S52.-), at wrist and hand level (ICD-10 S62.-), of lower leg, including ankle (ICD-10 S82.-).

The percentage of patients with any new incident fracture in the follow-up period as well as the number of fractures per patient and per observed patient-year (based on total follow-up time in the longitudinal cohort) were calculated; time to a new incident fracture was generated by a Kaplan-Meier estimator and depicted in a Kaplan-Meier curve. The estimation was censored for death or end of observation. The numbers were calculated separately for each type of new incident fracture and stratified by the type of index fracture.

#### Osteoporosis-related pharmacological treatment

OP-related drug treatment was described based on observed outpatient prescriptions in the follow-up periods and for the longitudinal cohort in the 12-month baseline period as well. Respective drugs have been identified via ATC code (Table 1 (Tab. 1)) as alendronic acid including combinations with calcium and/or colecalciferol, ibandronic acid incl. combinations with calcium and/or colecalciferol, risedronic acid incl. combinations with calcium and/or colecalciferol, zoledronic acid including combinations with calcium and/or colecalciferol, strontium ranelate, denosumab, teriparatide, raloxifene, estrogens and combinations.

All treatments with at least one respective prescription (only filled prescriptions available in the database) were reported. In the longitudinal analysis, the first agent prescribed within 90 days after the index fracture was defined as follow-up treatment. The treatment change between pre- and post-fracture was depicted in a Sankey diagram. Additionally, we calculated the number of patients who did not receive any OP treatment during the entire follow-up period. Due to their very restricted label, estrogens were excluded from our analysis.

#### HCRU

HCRU was assessed in terms of the number of patients with at least one OP-related outpatient visit (specialists and GPs, approximated by counted dates of invoiced codes according to the uniform valuation scheme (Einheitlicher Bewertungsmaßstab – EBM) and the related number of visits per patient and per patient-year (total follow-up time), as well as the number of patients with at least one inpatient stay and the respective number of inpatient visits per patient/patient-year and the number of days in the hospital per patient. We reported only OP-/fracture-related HCRU. Therefore, only GP/specialist visits related to an OP or fracture diagnosis (as previously defined) and hospitalizations with a main diagnosis of OP or fracture were taken into account.

#### Regulatory aspects and general considerations

As the study addressed a retrospective anonymized dataset, no ethical review and no informed consent of patients was needed. However, the study protocol was reviewed by a scientific steering committee and the data owner. The work on the dataset conformed to all social security data protection requirements.

Statistical analyses were performed using Microsoft SQL Server 2014, STATA/MP 14, and Microsoft Excel (MS 365).

## Results

### Patient selection and available observational periods

**Figure 1:**
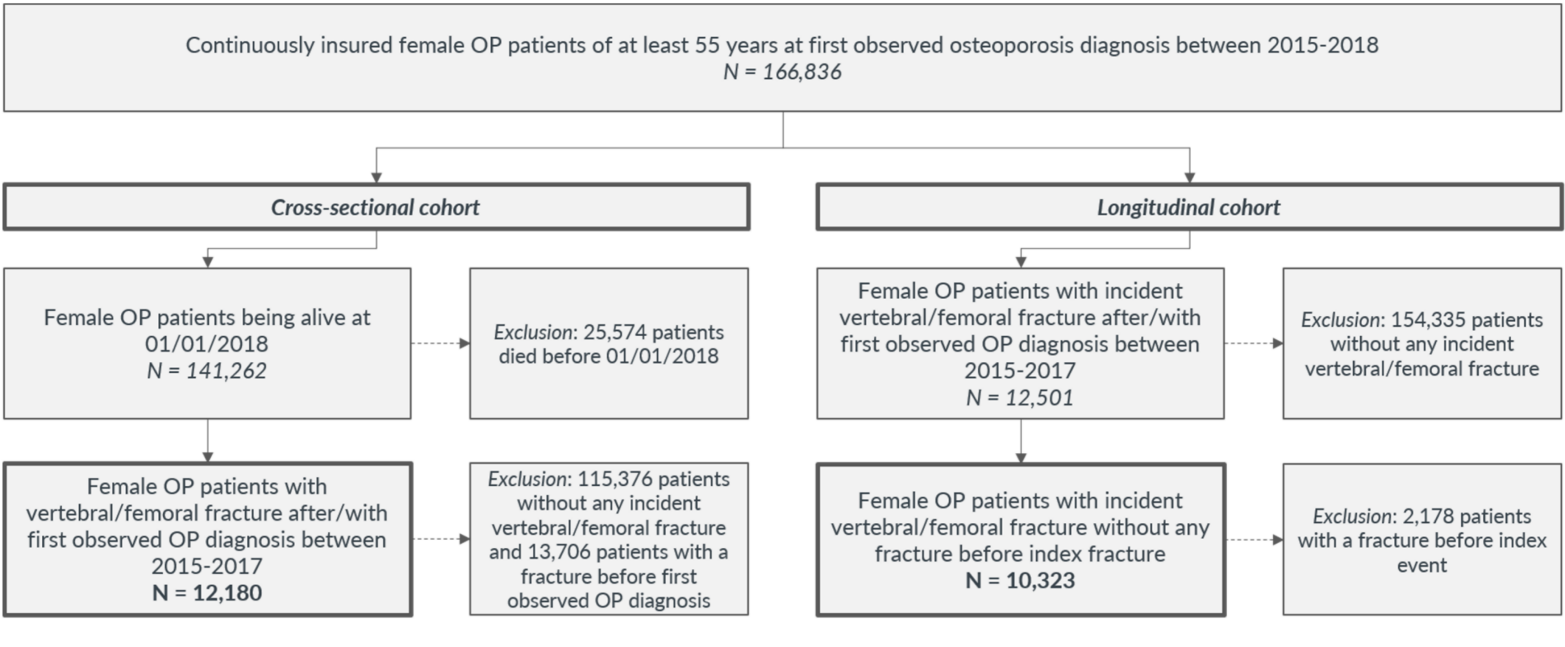
Patient attrition chart – inclusion and exclusion criteria for the cohort definitions

In the cross-sectional setting, we identified 12,180 female patients with an age of at least 55 years on 01/01/2018, an OP diagnosis between 01/01/2015–31/12/2017 and a vertebral or femoral fracture in 2015–2017 (Figure 1 (Fig. 1)). The mean observational time was 0.93 years in this cohort. 1,473 patients (12.09%) died in 2018 (0.130 deaths per patient-year).

For the longitudinal cohort, 10,323 female patients with an age of at least 55 years at the time of incident fracture could be identified (Figure 1 (Fig. 1)). The mean observational time was 2.03 years for these patients (range: 1–1,460 days). In this cohort, 3,387 patients (32.81%) died during follow-up (0.162 deaths per patient-year), and 537 patients (5.20%) died within the first 30 days after the incident fracture.

### Patient characteristics

On average, patients in the cross-sectional cohort were 83.59 years (SD: 8.24) old, with 64.71% in need for long-term care (Table 2 (Tab. 2)). The most frequently observed comorbidities were hypertension (86.80%), dorsalgia (53.02%), disorders of lipoprotein metabolism and other lipidaemias (49.20%), pain (44.25%), gonarthrosis (37.99%), and chronic ischaemic heart disease (36.68%). Eight hundred and forty patients (6.90%) experienced any of the investigated cardiovascular events in the pre-index period, with ischemic stroke being the most frequently observed event (2.41%), followed by angina pectoris (1.17%). Among the most frequently prescribed non-OP medications in the pre-index period were two pain medications: pyrazolones (58.49%) and other opioids (30.60%). Other frequently prescribed medications included proton pump inhibitors (55.73%), beta blocking agents (50.34%), and sulfonamides (43.30%).

Patients in the longitudinal cohort had a mean age of 83.22 years (SD: 8.17). In comparison to the cross-sectional cohort, a slightly lower proportion of patients were in need for long-term care (61.99%). The frequency and distribution of most often observed comorbidities, cardiovascular events, and non-OP medication were very similar to the cross-sectional sample (Table 2 (Tab. 2)), with some differences regarding percentage of patients with a pain diagnosis in the pre-index period (29.69%) and correspondingly lower rates of patients having received a pain medication (pyrazolones: 42.75%; other opioids: 23.22%).

### New incident fractures

In the cross-sectional sample, 1,742 patients (14.30%) sustained at least one new incident fracture in 2018 after their index fracture in 2015–2017. In total, 2,141 new incident fractures were identified, leading to an event rate of 0.19 fractures per patient-year. Two thirds of these fractures were vertebral or femoral fractures (1,501 fractures; 1,302 affected patients; 10.69%), and one third were other types of fractures (640 fractures; 582 affected patients; 4.78%).

In the longitudinal cohort, the most often observed index events were fractures of the femur (3,995 patients; 38.70%) and fractures of the lumbar spine or pelvis (3,593 patients; 34.81%), followed by fractures of the rib, sternum, or thoracic spine (2,283 patients; 22.12%). Six hundred and twenty-six patients (6.06%) had multiple fractures as index event.

**Figure 2:**
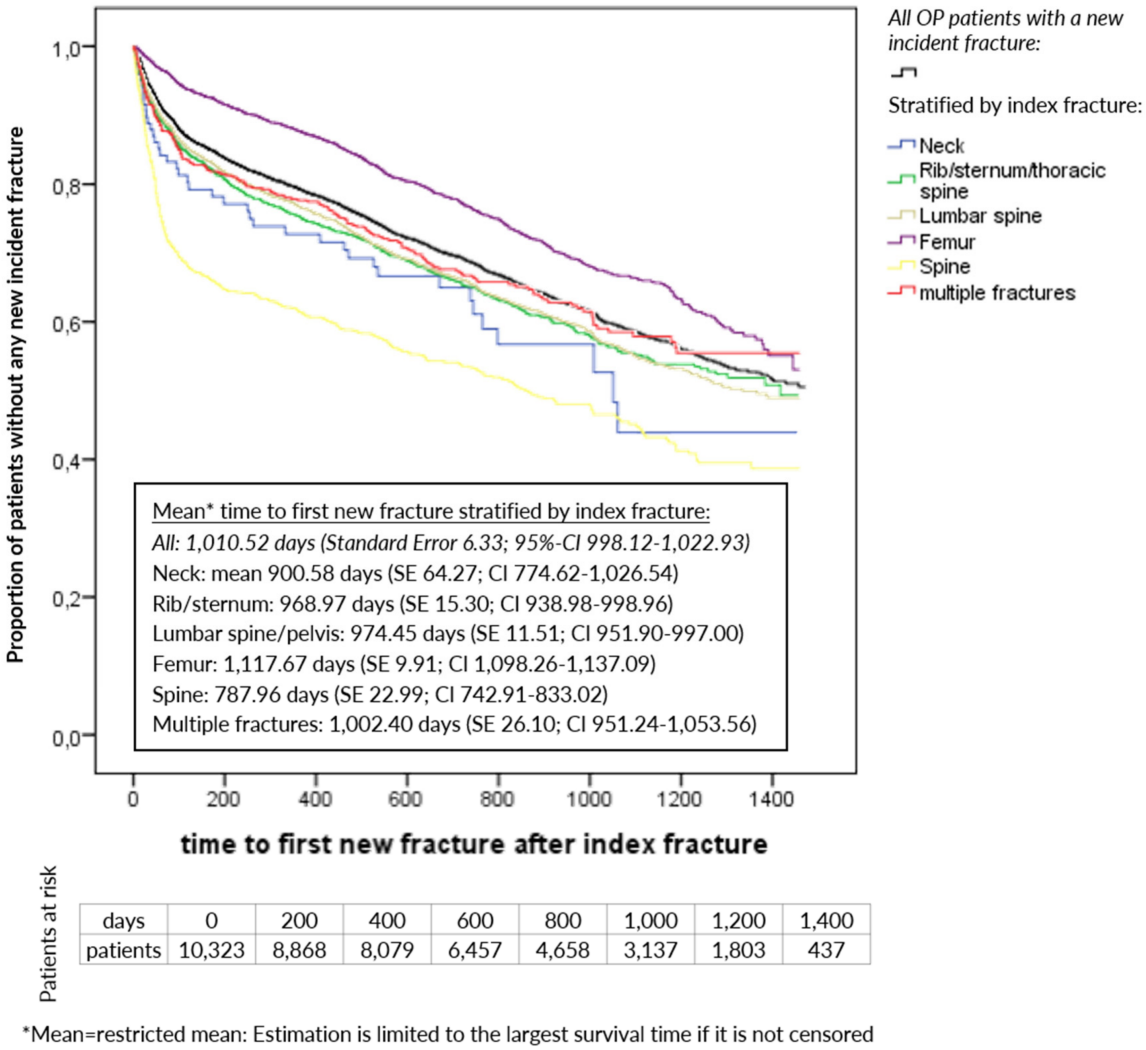
Kaplan-Meier curve: time to first new incident fracture stratified by index fracture (longitudinal cohort; censored for death or end of data availability)

Of all patients in the longitudinal cohort, 3,185 (30.85%) experienced at least one new incident fracture with an average of 1.39 fractures during the total follow-up time. In the first 12 months of the follow-up period, 1,957 patients (18.96%) were already affected by such a new incident fracture (Table 3 (Tab. 3)). Based on all patients, mean time to first new incident fracture was 1,010.52 days, ranging from 787.96 days for patients with a spine fracture as index event to 1,117.67 days for patients with a femur fracture as index event (Figure 2 (Fig. 2)).

### Treatment

In 2018, 34.54% of the cross-sectional cohort received a pharmaceutical OP treatment. The two most frequently prescribed medications were alendronic acid (14.27% of the patients) and denosumab (9.07%) (Table 4 (Tab. 4)).

**Figure 3:**
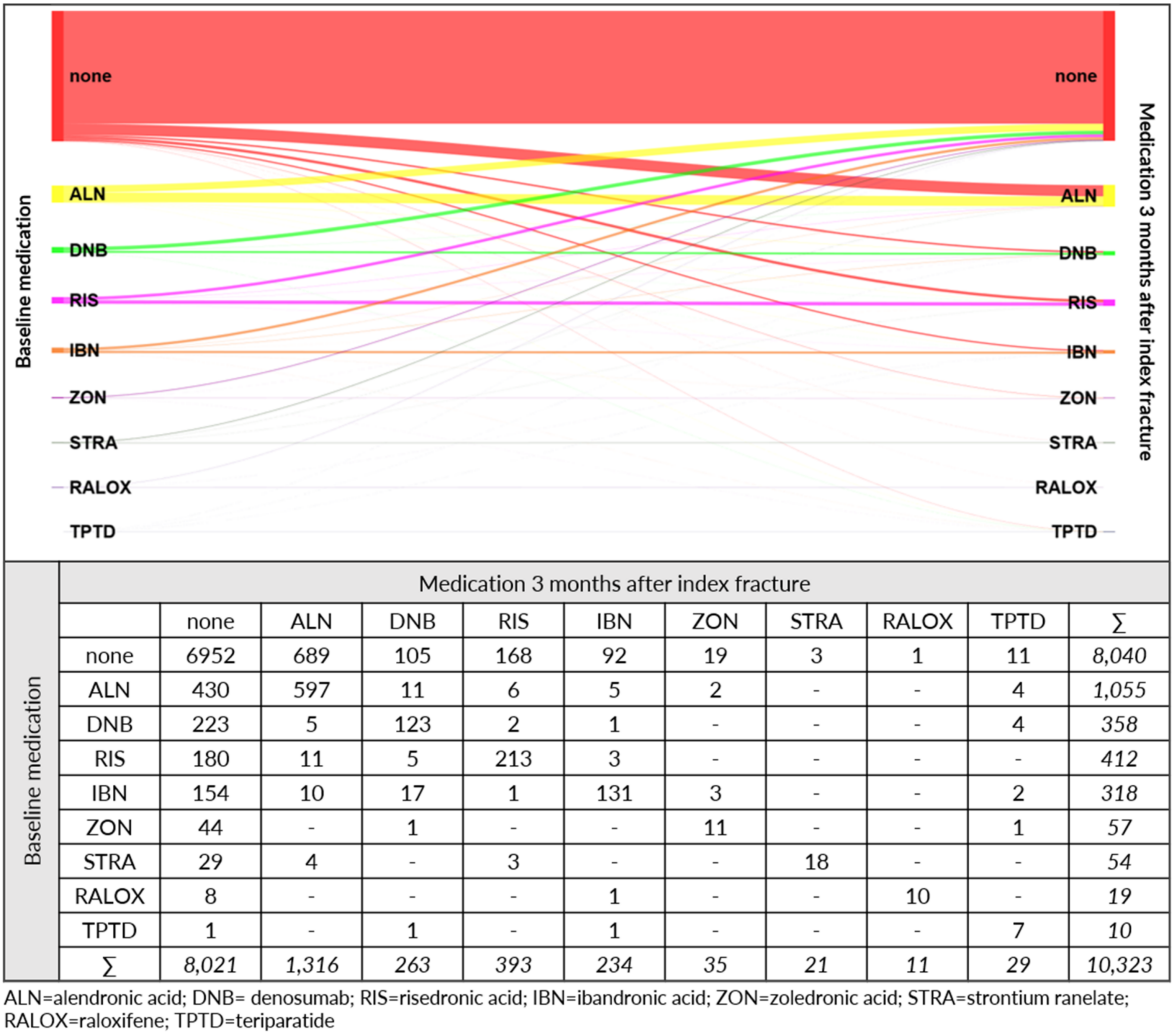
Sankey diagram for osteoporosis medication – number of patients with respective treatment before and after index fracture (longitudinal cohort)

Of the patients in the longitudinal cohort, 22.12% received an OP medication before the index fracture, with alendronic acid (10.22%), risedronic acid (3.99%), and denosumab (3.47%) as the most frequently prescribed agents (Figure 3 (Fig. 3)). Three months after the index fracture, 2,302 patients (22.30%) were prescribed with an OP treatment. Of the 2,283 patients with OP treatment during baseline, 1,214 patients also received medication after the index fracture. In total 6,952 patients (67.34%) did not receive any pharmaceutical OP treatment neither in the 12-month baseline period nor during the first 3 months after the index fracture.

The most frequently prescribed agent after the index fracture was alendronic acid (12.75%), followed by risedronic acid (3.81%), and denosumab (2.55%) (Figure 3 (Fig. 3)). Of all patients in the longitudinal cohort during the total follow-up time, 3,669 patients (35.54%) were prescribed with an OP treatment, 64.46% of the observed patients did not receive any OP medication at all.

### HCRU

Based on all patients in the cross-sectional cohort, 58.50% had at least one OP-related outpatient visit in 2018 (44.89% GP visits and 25.85% specialist visits; (Table 5 (Tab. 5)). Of all, 26.35% visited an outpatient physician at least once due to a fracture (13.17% GP and 13.40% specialists). At least one hospitalization related to OP or a fracture (main diagnosis) occurred in 160 patients (1.31%) and 1,293 patients (10.62%), with a mean length of stay of 14.27 days/15.90 days.

In the longitudinal cohort (Table 5 (Tab. 5)), patients had more fracture-related GP and specialist visits (24.54%/26.52% of the patients with at least one visit during the 12 months after the incident fracture, with a mean number of visits per patient-year of 0.61/0.57). The number of patients with OP-related outpatient visits during the first observed 12 months were comparable to the cross-sectional cohort (43.43% GP/25.62% specialists). Furthermore, 8.25% of patients had at least one OP-related hospitalization (mean duration: 14.99 days), and 8,473 patients (82.08%) had at least one fracture-related hospitalization (mean duration: 19.93 days) during the first 12 months after the incident fracture; 79.19% (6,710 patients) of these hospitalizations were related to the index fracture.

## Discussion

Based on a German large claims dataset, our study evaluated the real-world treatment and fracture rates of postmenopausal women with severe OP at high risk of fracture.

We observed two patient cohorts, one cross-sectional and one longitudinal. Whereas the purpose of the cross-sectional analysis was to observe the treatment of all severe OP patients at high risk of fracture in a more recent calendar year, the purpose of the longitudinal analysis was to observe treatment patterns and potential treatment changes after an incident fracture. To observe the entire year 2018/at least 12 months follow-up, OP patients with an incident fracture during 2018 were not included in either of the cohorts. However, due to the high age, high comorbidity burden, high level of care and the resulting high mortality of patients, 7,859 patients were included in both cohorts. Remaining differences between the cohorts, namely a slightly higher age and a higher level of needed care in the cross-sectional cohort, results from the fact that observation of longitudinal patients started immediately at the index fracture 2015–2017, whereas observation of cross-sectional patients might have started up to three years after the index fracture from 01/01/2015–31/12/2017.

Not in line with the recommendations of the German and European guidelines for the appropriate care of OP patients at high risk of fracture [3], [14], [15], about two-thirds of the observed patients in both of our cohorts (cross-sectional and longitudinal observation after incident fracture) did not receive any OP medication during the observed periods. Comparable results have been published in studies with European elderly women using different methodologies [21], [22], [23], [24]: The report of Hernlund et al. evaluated the burden of OP in the EU27 and concluded that the majority of individuals who have experienced an OP-related fracture or who are at high risk of fracture are untreated [21]. McCloskey et al. collected primary care data from women ≥70 years in a cross-sectional observational study in 8 European countries and found about 75% not receiving any OP medication despite their increased risk of fragility fracture [22].

We acknowledge that in addition to not prescribing the recommended OP medication, patient-driven non-persistence and non-adherence to OP medication are further reasons for a low real-world effectiveness of OP prophylaxis. In previous studies, 12-month persistence rates from 10.2% for oral bisphosphonates [25] to 85.3% for denosumab were reported [26]. A systematic review reported the proportion of patients adherent to oral bisphosphonates at 1 year between 31.7% and 72.0% [27].

We observed a considerable burden caused by fracture-related hospitalizations. Approximately 80% of observed patients experienced at least one fracture-related hospitalization in a 12-month follow-up. Our analysis additionally showed that 30% of patients with a previous vertebral/femoral fracture do experience another fracture during a mean follow-up of about 2 years, with nearly 20% of the patients having a recurrent fracture in the first 12 months after the incident fracture. Randomized controlled trials of approved OP medication (here: risedronate, raloxifene, and teriparatide) achieved 3-year fracture incidence rates in high-risk populations of 5–18% [28], [29], [30], [31]. This suggests that OP treatment, as recommended, could significantly reduce the fracture risk of these patients.

We acknowledge some limitations of our study. First, our data did not provide any information about clinical details such as bone mineral density (BMD) and vertebral trabecular bone score (TBS). Secondly, the used AOK PLUS dataset contains 3.4 million patients representing 4.7% of the German statutory health insurance population and covering only two specific regions of Germany (Saxony and Thuringia). Therefore, a regional bias of the study cannot be ruled out. Nevertheless, due to the fact that treatment patterns and reimbursement rules for statutory health insurances are comparable across Germany [32], the representativeness of the dataset concerning the diagnosis and treatments is likely. Thirdly, the used study methodology allows no detailed consideration of factors influencing patient-individual treatment decisions (for example, existing contraindications, the full clinical situation, treatment failure, or the occurrence of adverse events).

## Conclusions

Our study indicates that the real-world treatment of patients with severe OP at high risk of fracture can be improved. We observed a considerable proportion of untreated patients and a high rate of subsequent fractures. The impact of OP and related fractures on morbidity, mortality, and socioeconomic burden, especially in the geriatric population, is widely known. Therefore, the awareness for a proper risk assessment and the appropriate use of available treatments should be increased.

## Notes

### Competing interests

Antje Mevius participated in this study as staff member of IPAM and has nothing to disclose. Tanja Heidbrede, Patrick Gille and Hans Derk Pannen participated in the steering committee as staff members of UCB Pharma GmbH and have nothing to disclose. Thomas Wilke participated in this study as a staff member of IPAM and received honoraria from several pharmaceutical/consultancy companies (Novo Nordisk, Abbvie, Merck, GSK, BMS, LEO Pharma, Astra Zeneca, Bayer, Boehringer Ingelheim, Pharmerit).

### Funding

This research was funded by UCB Pharma and AMGEN in accordance with Good Publication Practice (GPP3) guidelines.

## Figures and Tables

**Table 1:**
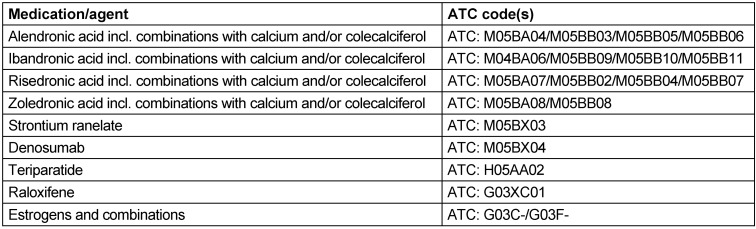
ATC codes of OP medication

**Table 2:**
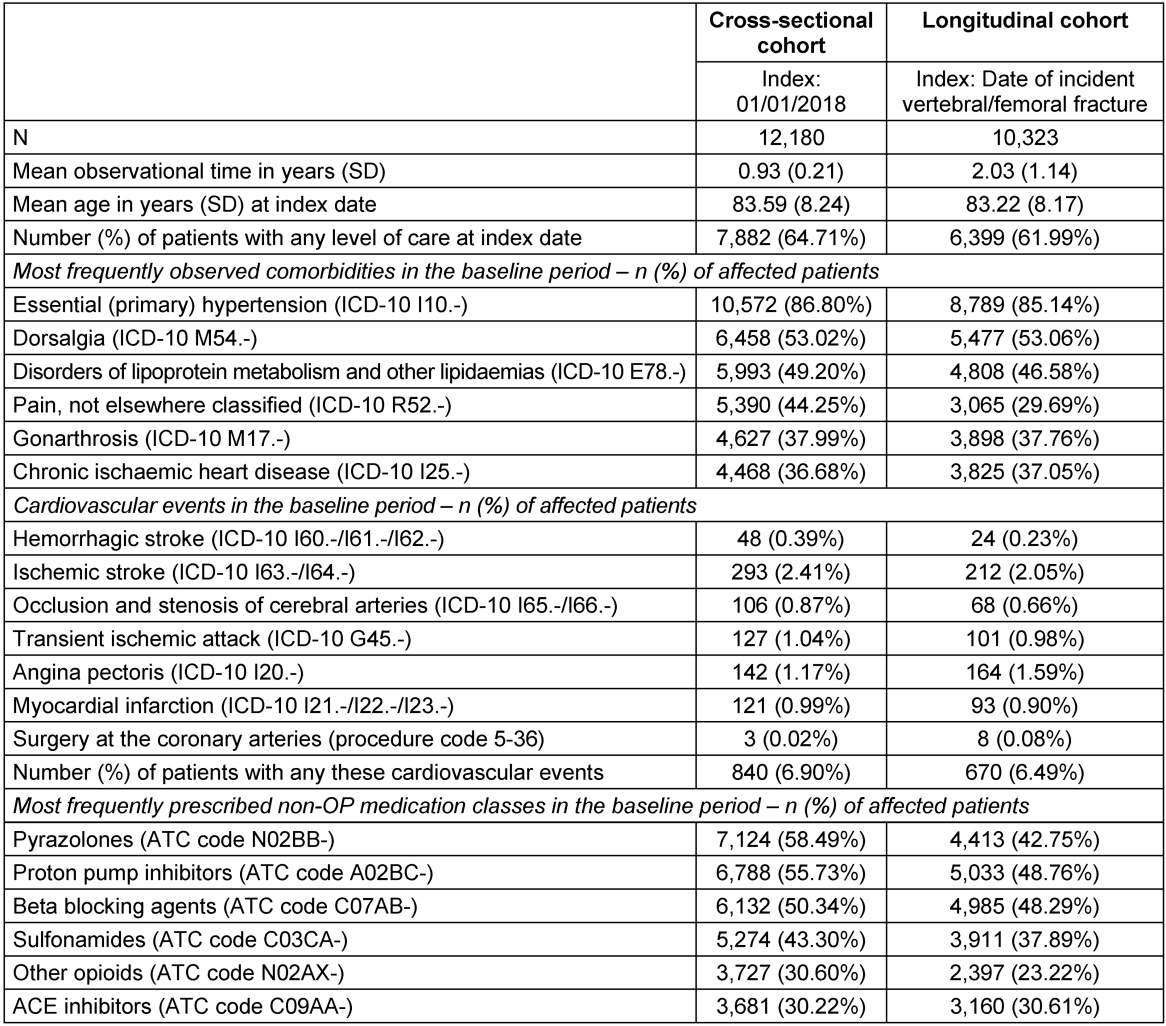
Baseline characteristics of analyzed cohorts

**Table 3:**
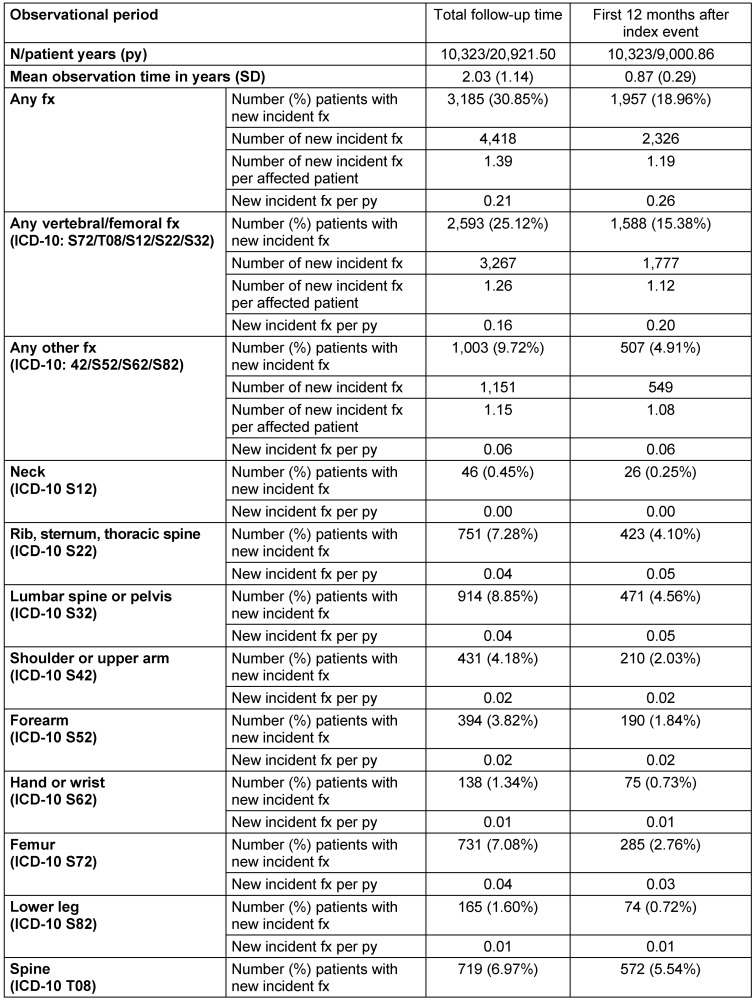
New incident fractures (fx) during follow-up after index fracture (longitudinal cohort)

**Table 4:**
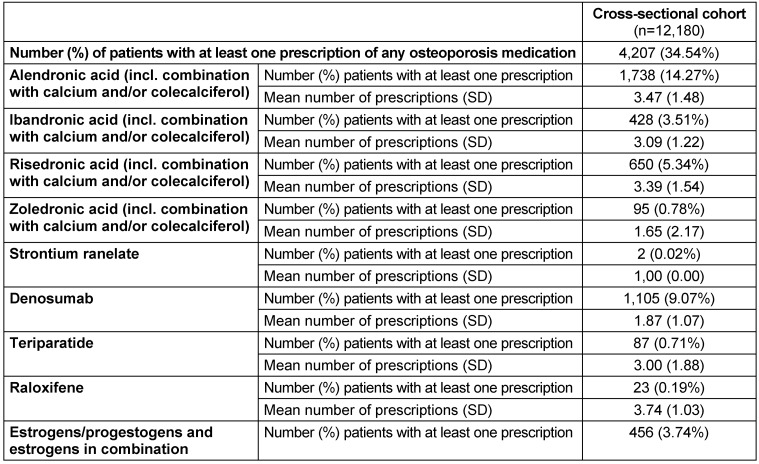
Osteoporosis medication treatment in 2018 (cross-sectional sample)

**Table 5:**
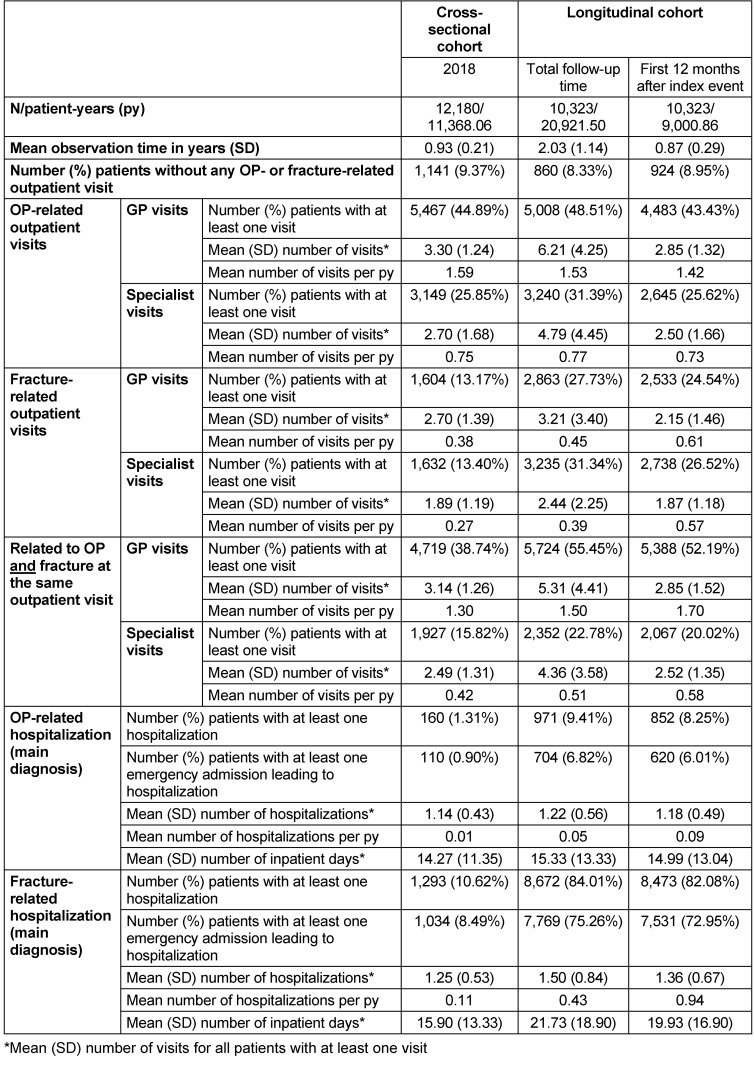
HCRU during follow-up
